# Biosynthesis and Genetic Encoding of Non-hydrolyzable Phosphoserine into Recombinant Proteins in *Escherichia coli*

**DOI:** 10.21769/BioProtoc.4861

**Published:** 2023-11-05

**Authors:** Phillip Zhu, Ryan A. Mehl, Richard B. Cooley

**Affiliations:** 1 Department of Biochemistry and Biophysics, Oregon State University, 2011 Agricultural and Life Sciences, Corvallis, OR, USA; 2 GCE4All Research Center, Oregon State University, 2011 Agricultural and Life Sciences, Corvallis, OR, USA

**Keywords:** Phosphoserine, Non-hydrolyzable phosphoserine, Genetic code expansion, Amber suppression, Biosynthetic pathway, Recombinant protein expression, PermaPhos

## Abstract

While site-specific translational encoding of phosphoserine (pSer) into proteins in *Escherichia coli* via genetic code expansion (GCE) technologies has transformed our ability to study phospho-protein structure and function, recombinant phospho-proteins can be dephosphorylated during expression/purification, and their exposure to cellular-like environments such as cell lysates results in rapid reversion back to the non-phosphorylated form. To help overcome these challenges, we developed an efficient and scalable *E. coli* GCE expression system enabling site-specific incorporation of a non-hydrolyzable phosphoserine (nhpSer) mimic into proteins of interest. This nhpSer mimic, with the γ-oxygen of phosphoserine replaced by a methylene (CH_2_) group, is impervious to hydrolysis and recapitulates phosphoserine function even when phosphomimetics aspartate and glutamate do not. Key to this expression system is the co-expression of a *Streptomyces* biosynthetic pathway that converts the central metabolite phosphoenolpyruvate into non-hydrolyzable phosphoserine (nhpSer) amino acid, which provides a > 40-fold improvement in expression yields compared to media supplementation by increasing bioavailability of nhpSer and enables scalability of expressions. This “PermaPhos” expression system uses the E. coli BL21(DE3) Δ*serC* strain and three plasmids that express (i) the protein of interest, (ii) the GCE machinery for translational installation of nhpSer at UAG amber stop codons, and (iii) the *Streptomyces* nhpSer biosynthetic pathway. Successful expression requires efficient transformation of all three plasmids simultaneously into the expression host, and IPTG is used to induce expression of all components. Permanently phosphorylated proteins made in *E. coli* are particularly useful for discovering phosphorylation-dependent protein–protein interaction networks from cell lysates or transfected cells.

Key features

• Protocol builds on the nhpSer GCE system by Rogerson et al. (2015), but with a > 40-fold improvement in yields enabled by the nhpSer biosynthetic pathway.

• Protein expression uses standard Terrific Broth (TB) media and requires three days to complete.

• C-terminal purification tags on target protein are recommended to avoid co-purification of prematurely truncated protein with full-length nhpSer-containing protein.

• Phos-tag gel electrophoresis provides a convenient method to confirm accurate nhpSer encoding, as it can distinguish between non-phosphorylated, pSer- and nhpSer-containing variants.

**Graphical overview**


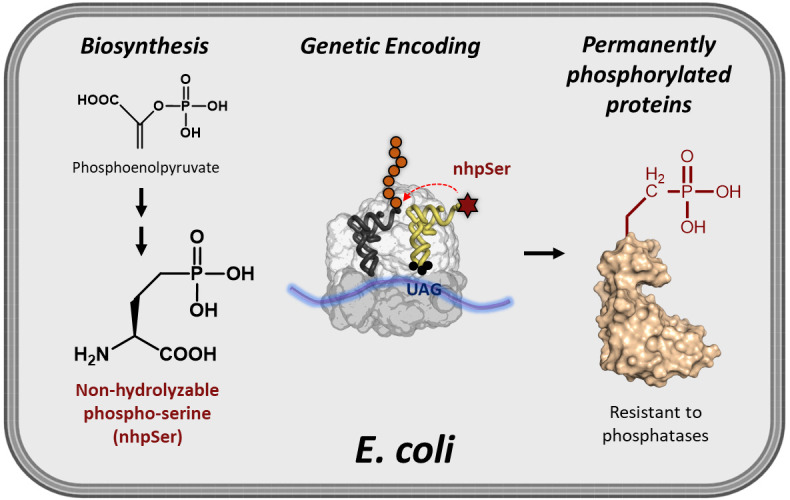


## Background

Discerning molecular mechanisms by which phosphorylation alters protein structure and function requires efficient methods to make homogenously and site-specifically phosphorylated proteins in milligram quantities for in vitro characterization. Kinases can be used to install phospho-groups onto target proteins, but the complexities of kinase specificity and their activation hinder broad application of these approaches. Genetic code expansion (GCE), on the other hand, enables production of site-specifically phosphorylated proteins by installing phospho-amino acids into proteins during translation in response to UAG (amber) stop codons. *Escherichia coli* GCE systems for expressing proteins homogenously modified with one or more phosphoserine (pSer) groups are well developed and becoming more widely used (Rogerson et al., 2015; Zhu et al., 2019). However, pSer proteins produced in *E. coli* via these GCE encoding systems can become partially dephosphorylated during expression by a few phosphatases that *E. coli* does harbor, frustrating downstream applications. Further, experiments requiring exposure of purified phospho-proteins to environments with phosphatases will compromise their phosphorylation status. As one example where this is an issue, “pulldowns” are a time-tested technique to discover new protein–protein interactions whereby *bait* proteins are mixed into cell lysates and then retrieved along with any bound *prey* proteins, which can then be identified by mass spectrometry or immunological assays. A phosphorylated bait protein will be hydrolyzed back to the wild-type form by phosphatases when added to eukaryotic lysates, even in the presence of phosphatase inhibitors, so that the retrieved pool of prey proteins will be a mixture of wild-type and phospho-protein interactors (Zhu et al., 2023). Consequently, it is challenging (if not impossible) to distinguish between proteins that preferentially interact with the phosphorylated or the wild-type form of the prey protein.

**Figure 1.**
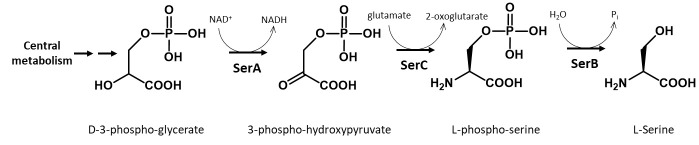
Biosynthesis of serine in *E. coli*. In this protocol, a ∆*serC* mutant expression host is used as it lacks the ability to biosynthesize pSer amino acid, which would compete with nhpSer encoding. SerB is also overexpressed to hydrolyze any free pSer amino acid that might enter the cell from the media or be formed by promiscuous transaminases that can substitute for SerC function. For GCE systems that encode authentic phosphoserine, ∆*serB* expression hosts are used in order to build up intracellular pSer concentrations.

**Figure 2.**
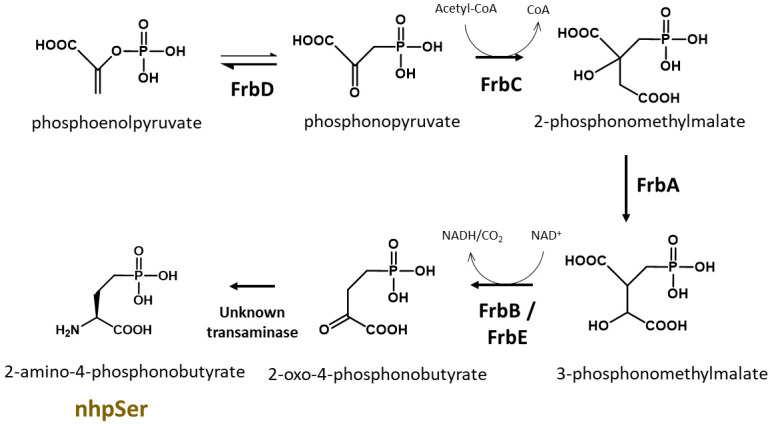
Biosynthesis of nhpSer amino acid by *Streptomyces rubellomurinus* FrbA, B, C, D, and E proteins. In this protocol, the pCDF-Frb-v1 plasmid expresses five Frb enzymes that, along with an unknown transaminase in *E. coli*, convert the central metabolite phosphoenolpyruvate into nhpSer, which is then encoded into target proteins using the GCE machinery.

A GCE system to encode a stable, non-hydrolyzable mimic of pSer called phosphono-methyl-alanine (also known as 2-amino-4-phosphono-butryate and here referred to as non-hydrolyzable pSer or “nhpSer” for simplicity) was developed by Chin and colleagues in 2015 (Rogerson et al., 2015). This system relies on adding exogenous nhpSer to the culture media while expressing the same amino-acyl tRNA synthetase (RS)/tRNA pair and EF-Tu variant (EF-Sep) used for pSer incorporation. To ensure nhpSer is encoded over endogenous pSer, an *E. coli* Δ*serC* mutant is used in combination with overexpression of SerB (Figure 1). The negatively charged nhpSer does not traverse the cellular membrane effectively when exogenously added to the culture media, and so we developed a 6-step biosynthetic pathway that converts phosphoenolpyruvate into nhpSer inside the cell (Figure 2) to increase bioavailability of nhpSer for subsequent encoding into a protein, providing > 40-fold improvement in nhpSer-protein production and enabling scalability of expressions (Zhu et al., 2023). This “PermaPhos” expression system provided us with sufficient quantities of nhpSer-containing proteins to confirm that nhpSer mimics pSer function in several cases where aspartate/glutamate do not (Zhu et al., 2023). We outline here the general workflow for expressing a control protein (super folder GFP, sfGFP) containing nhpSer at site N150, and confirming correct encoding. Strategies to adopt this protocol for biologically relevant proteins are discussed.

## Materials and reagents

**Biological materials**

**Strains**

1. BL21(DE3) ∆*serC* (Addgene, #197656). This BL21(DE3) strain of *E. coli* has the *serC* gene knocked out to prevent biosynthesis of phosphoserine, which would compete with nhpSer for incorporation into proteins (Figure 1). This strain contains Release Factor 1 (RF1), the protein responsible for terminating translation at TAG amber codons, so truncated protein will be produced along with full-length nhpSer-protein. To avoid co-purification of truncated protein with full-length protein, C-terminal purification tags are recommended. For proteins that self-assemble into homo-multimers (dimers, trimers, etc.), purification can be challenging due to the possible co-purification of truncated forms that are incorporated as subunits in the assembly. If using N-terminal purification/solubilization tags, additional purifications steps may be needed to remove truncated protein species. See General note 1 for more discussion.
2. DH10b (Thermo Fisher, catalog number: EC0113). This strain can be used for faithful propagation of plasmids and for cloning needs when users wish to clone their genes of interest into the pRBC plasmid (see Plasmids below). Do not use for protein expression. Though we have not explicitly tested all of them, other classical cloning strains of *E. coli* can be used including NEB 10-beta (New England BioLabs, catalog number: C3019H), DH5α (e.g., Thermo Fisher, catalog number: 18258012), NEB 5-alpha (New England BioLabs, catalog number: C2987H), or TOP10 (Thermo Fisher, catalog number: C404010).

**Plasmids**

1. pERM2-nhpSer (Addgene, #201922): machinery plasmid for nhpSer incorporation, kanamycin resistance with pUC origin of replication. This plasmid expresses the same tRNA synthetase and EF-Tu used for pSer incorporation (Rogerson et al., 2015). The amber codon suppressing Sep-tRNA_CUA_ is v2.0 developed by Chin and colleagues to minimize mis-aminoacylation (Zhang et al., 2017). The Sep-tRNA and tRNA-synthetase are constitutively expressed via lpp and GlnS promoters, respectively. The EF-Tu is under control of an IPTG inducible tac promoter. Also constitutively expressed via an OXB20 promoter is the *E. coli* serB protein to further eliminate (hydrolyze) free pSer amino acid that may come from the media or produced by promiscuous transaminases.
2. pCDF-Frb-v1 (Addgene, #201923): expresses *Streptomyces rubellomurinus* FrbA, FrbB, FrbC, FrbD, and FrbE enzymes required for the biosynthesis of nhpSer. See Zhu et al. (2023) for a detailed description of this biosynthetic pathway, which is also summarized in Figure 2. Each Frb protein is expressed under the control of an engineered T7 transcriptional promoter variant and so all Frb enzyme are expressed by the addition of IPTG. This plasmid confers spectinomycin resistance and contains the CloDF origin of replication. This plasmid is large (~11 kb) and contains repetitive elements; while we have not observed instability of this plasmid, given its large size it is good practice to minimize propagations. We recommend that when you receive the DH10b cells with the pCDF-Frb-v1 plasmid from Addgene, grow up a few individual colonies overnight in liquid media [e.g., 2× YT media supplemented with spectinomycin at 100 μg/mL (see Recipes)] and make frozen glycerol stocks of the cells for long-term storage. Glycerol stocks can be made by mixing 600 μL of overnight culture with 400 μL of sterile 50% (v/v) glycerol. Place culture tube(s) in -80 °C freezer for storage. These stocks serve as a permanent, long-term source of plasmid, which can be prepared by inoculating cultures with cells from a glob of the frozen glycerol stock. Do not thaw the glycerol stock(s) once frozen.
3. pRBC-sfGFP wild-type (Addgene, #174075): expresses wild-type sfGFP control protein with C-terminal His6 tag, under a T7 transcriptional promoter, ampicillin resistance/p15a origin of replication. sfGFP is expressed by the addition of IPTG.
4. pRBC-sfGFP 150TAG (Addgene, #174076): same as above except the sfGFP gene contains a TAG amber stop codon at site N150. The TAG codon is used to direct the translational encoding of nhpSer. Note that this is also the same plasmid used for pSer encoding (Zhu et al., 2022); any protein cloned into this vector for pSer encoding will also work for nhpSer encoding. See General note 2 for more discussions.
5. pRBC-[x] wild type (you must create): expresses your wild-type protein of interest (POI). You can clone your POI into pRBC by removing the sfGFP gene from the pRBC-sfGFP wt plasmid by restriction digest with NdeI and XhoI enzymes and replacing it with your POI gene using standard cloning techniques (e.g., ligation, Gibson Assembly, or SLiCE). For reasons mentioned above, a C-terminal purification tag is preferred. For helpful tips on construct design, see Troubleshooting 1.
6. pRBC-[x] TAG (you must create): expresses your POI with nhpSer encoded at a TAG codon. You can clone your POI into pRBC by removing the sfGFP gene by restriction digest with NdeI and XhoI and replacing with your POI gene using standard cloning techniques (e.g., ligation, Gibson Assembly, or SLiCE). Using site-directed mutagenesis, change the codon to TAG (the amber stop codon) where you intend to encode nhpSer into your POI.

**Reagents**

1. Tryptone (e.g., VWR, catalog number: 97063-386)
2. Yeast Extract (e.g., VWR, catalog number: 97064-368)
3. NaCl (e.g., VWR, catalog number: 97061-274)
4. Agar (e.g., VWR, catalog number: 97064-336)
5. MgSO_4_·7H_2_O (e.g., VWR, catalog number: 97062-134)
6. K_2_HPO_4_ (potassium dibasic) (e.g., VWR, catalog number: 97062-234)
7. KH_2_HPO_4_ (potassium monobasic) (e.g., VWR, catalog number: BDH9268)
8. α-D-glucose (e.g., VWR, catalog number: 97061-168)
9. Glycerol (e.g., VWR, catalog number: BDH24388.320)
10. Isopropyl b-D-1-thiogalactopyranoside (IPTG) (e.g., Anatrace, catalog number: I1003)
11. Ampicillin (e.g., VWR, catalog number: 97061-442)
12. Kanamycin (e.g., VWR, catalog number: 75856-684)
13. Spectinomycin (e.g., VWR, catalog number: 89156-368)
14. Antifoam B (e.g., J.T. Baker, catalog number: B531-05)
15. Phos-tag acrylamide (e.g., VWR, catalog number: 101974-086)
16. Dry ice
17. Ethanol (e.g., Sigma, catalog number: 65348-M)
18. Acrylamide/Bio-acrylamide solution (e.g., Bio-Rad, catalog number: 1610146)

**Solutions**

1. Phosphate Buffered Saline (PBS) (e.g., VWR, catalog number: 75800-982)
2. LB/agar (see Recipes)
3. 2× YT media (see Recipes)
4. 50% (v/v) glycerol (see Recipes)
5. 10% (v/v) glycerol (see Recipes)
6. SOC media (see Recipes)
7. Starter culture media (see Recipes)
8. 1.1× Terrific Broth media (see Recipes)
9. 10× TB potassium phosphate buffer (see Recipes)
10. Ampicillin stock (see Recipes)
11. Kanamycin stock (see Recipes)
12. Spectinomycin stock (see Recipes)
13. 0.5 M IPTG (see Recipes)

**Recipes**

1. **LB/agar media (0.5 L)**
  **Table:** 

Reagent	Final concentration	Quantity
Tryptone	1% (w/v)	5 g
Yeast extract	0.5% (w/v)	2.5 g
NaCl	1.0% (w/v)	5 g
Agar	1.5% (w/v)	7.5 g
H_2_O	n/a	To 500 mL
Total	n/a	500 mL
  - After mixing reagents thoroughly, autoclave on standard liquid setting to sterilize. Note the agar will not go into solution until autoclaved.
  - After autoclaving, gently swirl the bottle to ensure melted agar is evenly mixed.
*Notes:*
*i. Store LB/agar bottle in a 55 °C oven and pour plates on an as needed basis. LB/agar can be stored in molten form for ~2 weeks if sterility is maintained.*
*ii. If an oven is not available, plates can be poured with antibiotics once LB/agar is sufficiently cooled to touch. Plates can be stored at 4 °C for up to a week.*
2. **2× YT media (1 L)**
  **Table:** 

Reagent	Final concentration	Quantity
Tryptone	1.6% (w/v)	16 g
Yeast extract	1.0% (w/v)	10 g
NaCl	0.5% (w/v)	5 g
H_2_O	n/a	To 1,000 mL
Total	n/a	1 L
  - After mixing reagents thoroughly, autoclave on the standard liquid setting to sterilize.
  - After autoclaving, allow to cool to room temperature before use.
3. **50% (v/v) glycerol (500 mL)**
  **Table:** 

Reagent	Final concentration	Quantity
Glycerol (100%)	50% (v/v)	250 mL
H_2_O	n/a	250 mL
Total	n/a	500 mL
  Mix by placing a suitable magnetic stir bar in a 500 mL graduated cylinder and add 250 mL of water to graduated cylinder. While stirring, pour pure glycerol to the 500 mL mark on graduated cylinder. Stir for 5 min and then transfer to a 0.5 L bottle. Sterilize by autoclaving on liquid setting.
4. **10% (v/v) glycerol (1 L)**
  **Table:** 

Reagent	Final concentration	Quantity
Glycerol (100%)	10% (v/v)	100 mL
H_2_O	n/a	900 mL
Total	n/a	1 L
  Mix by placing a suitable magnetic stir bar in a 1,000 mL graduated cylinder and add 900 mL of water to graduated cylinder. While stirring, pour pure glycerol to the 1,000 mL mark on graduated cylinder. Stir for 5 min and then transfer to a 1 L bottle. Sterilize by autoclaving on liquid setting.
5. **SOC media, 50 mL**
  **Table:** 

Reagent	Final concentration	Quantity
2× YT media	n/a	49 mL
1 M MgSO_4_	10 mM	0.5 mL
40% (w/v) α-D-glucose	0.4% (w/v) or ~20 mM	0.5 mL
Total	n/a	50 mL
  - 1 M MgSO_4_ can be made by mixing 12.3 g of MgSO_4_·7H_2_O in water up to 50 mL of total volume. Adjust mass of MgSO_4_ if using the salt with different hydration status.
  - 40% (w/v) α-D-glucose can be made by mixing 20 g of α-D-glucose with water up to 50 mL of total volume. Mix thoroughly until glucose is dissolved. Gentle heating in a microwave may facilitate dissolution of glucose.
  - Sterilize MgSO_4_, glucose, and 2× YT solutions individually by autoclaving. Allow each component to cool to room temperature and mix as indicated above. Maintain sterility while adding components together.
  - It is easy to contaminate SOC. We suggest breaking this into 5 × 10 mL aliquots before use or making smaller batches. If sterility is maintained, SOC can be stored at room temperature indefinitely. It can also be stored at -20 °C but avoid repeated freeze/thaw.
6. **Starter culture media: 2× YT + 0.5% (v/v) glycerol (50 mL)**
  **Table:** 

Reagent	Final concentration	Quantity
2× YT media (sterile)	n/a	49.5 mL
50% (v/v) glycerol (sterile)	0.5% (v/v)	0.5 mL
Total	n/a	50 mL
  Prepare immediately before use.
7. **1.1× Terrific Broth media (0.9 L)**
  **Table:** 

Reagent	Final concentration (of 1×)	Quantity
Tryptone	1.2% (w/v)	12 g
Yeast extract	2.4% (w/v)	24 g
Glycerol (50% [v/v])	0.5% (v/v)	10 mL
H_2_O	n/a	To 900 mL
Total	n/a	900 mL
  - Sterilize by autoclaving on liquid setting.
  - Do not mix TB phosphate buffer (below) until all solutions are cool and immediately before use.
8. **10× TB potassium phosphate buffer (1 L)**
  **Table:** 

Reagent	Final concentration	Quantity
KH_2_PO_4_ (monobasic, anhydrous)	0.17 M	23.1 g
K_2_HPO_4_ (dibasic, anhydrous)	0.72 M	125.4 g
H_2_O	n/a	To 1 L
Total	n/a	1 L
  - The masses provided for the potassium phosphate salts are for anhydrous formulations. Hydrate forms of these salts can be used, but masses must be adjusted to maintain indicated molarity.
  - Sterilize by autoclaving on liquid setting.
  - Do not mix TB phosphate buffer to 1.1× TB media until all solutions are sterilized and cooled to room temperature, and immediately before use.
9. **Ampicillin stock (10 mL)**
  **Table:** 

Reagent	Final concentration	Quantity
Ampicillin	100 mg/mL	1 g
H_2_O	n/a	To 10 mL
Total	n/a	10 mL
  - Sterilize by filtering with a 0.2 μm syringe-end filter.
  - Store in 1 mL aliquots at -20 °C.
10. **Kanamycin stock (10 mL)**
  **Table:** 

Reagent	Final concentration	Quantity
Kanamycin	50 mg/mL	0.5 g
H_2_O	n/a	To 10 mL
Total	n/a	10 mL
  - Sterilize by filtering with a 0.2 μm syringe-end filter.
  - Store in 1 mL aliquots at -20 °C.
11. **Spectinomycin stock (10 mL)**
  **Table:** 

Reagent	Final concentration	Quantity
Spectinomycin	100 mg/mL	1 g
H_2_O	n/a	To 10 mL
Total	n/a	10 mL
  - Sterilize by filtering with a 0.2 μm syringe-end filter.
  - Store in 1 mL aliquots at -20 °C.
*Note: Do not confuse spectinomycin with streptomycin. These antibiotics are not interchangeable.*
12. **0.5 M IPTG (10 mL)**
  **Table:** 

Reagent	Final concentration	Quantity
IPTG	0.5 M	1.19 g
H_2_O	n/a	To 10 mL
Total	n/a	10 mL
  - Sterilize by filtering with a 0.2 μm syringe-end filter.
  - Store in 1 mL aliquots at -20 °C.
**Laboratory supplies**
  1. 1.7 mL Eppendorf tubes (e.g., VWR, catalog number: 87003-294)
  2. 0.6 mL Eppendorf tubes (e.g., VWR, catalog number: 89000-010)
  3. 100 mm plates (e.g., VWR, catalog number: 470210-568)
  4. 0.5 L centrifuge bottles (must fit available centrifuges/rotor)
  5. 500 mL graduate cylinder
  6. 15 mL conical tubes (e.g., VWR, catalog number: 89126-798)
  7. 50 mL conical tubes (e.g., VWR, catalog number: 89039-656)
  8. 14 mL sterile culture tubes (e.g., VWR, catalog number: 60818-689)
  9. 250 mL baffled flasks (e.g., VWR, catalog number: 89095-266)
  10. 2.8 L baffled Fernbach flasks (e.g., VWR, catalog number: 22877-168)
  11. Micro pipette tips 10 μL (e.g., VWR, catalog number: 76323-394)
  12. Micro pipette tips 200 μL (e.g., VWR, catalog number: 76323-390)
  13. Micro pipette tips 1,000 μL (e.g., VWR, catalog number: 76323-454)
  14. 0.2 μm syringe end filter (e.g., VWR, catalog number: 28145-477)
  15. 10 mL syringes (e.g., VWR, catalog number: 76124-664)

## Equipment

1. Autoclave (any capable of sterilizing liquid media and culturing materials at 121 °C, and of a saturated steam pressure of 15 PSI)
2. Expression equipment:
  - Static incubator for growing LB/agar plates (set to 37 °C) (e.g., VWR, catalog number: 97025-630)
  - Shaker incubator for growing liquid cultures (e.g., New Brunswick I26R, Eppendorf, catalog number: M1324-0004)
    i. Shaker should be able to rotate at 200–250 rpm
    ii. Refrigeration is necessary for expressions below room temperature (< 25 °C)
    iii. Shaker deck should have clamps to hold 250 mL and 2.8 L Fernbach flasks
  - Optical density 600 nm spectrophotometer (e.g., Ultrospec 10, Biochrome, catalog number: 80-2116-30)
3. Competent cell preparation and cell harvesting
  - Centrifuge capable of speeds of at least 5,000× *g* and able to hold volumes commensurate with culture sizes (e.g., Beckman Coulter, model: Allegra 25R)
  - Centrifuge bottles that can withstand centrifugal forces and hold the volume of cultures grown for harvesting cells. The type of bottles depends on the centrifuge and rotor used
  - Freezer (-80 °C), if cells are to be stored before protein is purified (e.g., VIP ECO ULT freezer, VWR, catalog number: 76305-596)
4. Electroporator (e.g., Eppendorf Eporator, Fisher Scientific, catalog number: E4309000027)
5. 0.1 cm electro-cuvettes (e.g., Bio-Rad, catalog number:1652089)
6. Fluorometer capable of reading sfGFP fluorescence (excitation 488 nm/emission 512 nm). Handheld fluorometers work well for routine fluorescence reads (e.g., Turner Biosystems, model: PicoFluor)
-20 °C freezer for storing plasmids and antibiotics (e.g., Fisher Scientific, catalog number: 10-549-264)
8. Ice machine (e.g., Fisher Scientific, catalog number: 09-540-003)

## Procedure

In Part A, you first prepare electro-competent BL21(DE3) ∆*serC* cells before expressing protein in Part B. These electro-competent cells need to be highly efficient for expressions to be successful (see Troubleshooting 2). The process outlined below is sufficient to produce enough of electro-competent cells for ~20–30 transformations and expressions.

- **Preparation of electro-competent BL21(DE3) ∆*serC* cells**
  1. Day 1
    - Prepare LB/agar plate (no antibiotics) and cool to room temperature to solidify.
    - Streak out BL21(DE3) ∆*serC* cells onto LB/agar plate from a small glob of cells taken from a frozen glycerol stock.
    - Place LB/agar plate with streaked cells upside down in a static 37 °C incubator and grow overnight (14–20 h).
  2. Day 2
    - After overnight growth, place LB/agar plate with BL21(DE3) ∆*serC* cells in a refrigerator until the end of the day.
    - Prepare and autoclave 1 L of 2× YT media in a 2.8 L baffled Fernbach flask.
    - Prepare 1 L of 10% (v/v) glycerol and sterilize by autoclaving along with the following:
      i. 500 mL graduated cylinder.
      ii. 2 × 0.5 L centrifuge bottles (or bottles suitable for centrifuging 1 L of culture at 4,000× *g*). Centrifuge bottles should be thoroughly cleaned to remove any residual DNA or cells that could contaminate electrocompetent cells prior to autoclaving.
      iii. A box each of 1,000, 100, and 10 μL pipette tips.
      iv. Approximately fifty 0.6 mL Eppendorf tubes.
    - After autoclaving, place the 10% (v/v) glycerol solution in a refrigerator or cold room to chill overnight.
    - At the end of the day, add 5 mL of 2× YT media to a 14 mL culture tube and inoculate with a single colony of BL21(DE3) ∆*serC* cells grown the prior night on the LB/agar plate. Grow liquid culture at 37 °C overnight with shaking at 250 rpm.
  3. Day 3
    - Inoculate 1 L of 2× YT media (in a 2.8 L baffled Fernbach flask) with the 5 mL of overnight starter culture.
      Add ~5–6 drops of anti-foam to the culture flask. Excessive foaming of the media caused by the flask baffles with inhibit air exchange and result in slower cell growth.
    - Grow the 1 L culture at 37 °C with shaking at 200–250 rpm until OD_600_ ~0.4. This should take approximately 2–3 h. Do not let culture grow above OD_600_ ~0.5. It is a good idea to check the OD_600_ approximately 1.5 h after inoculation to verify if cells are growing.
    - While cells are growing, pre-chill centrifuge and rotor to 4 °C and place centrifuge bottle(s) and graduated cylinder in refrigerator to chill as well.
    - Once cells reach OD_600_ ~0.4, immediately place culture flask in an ice/water bath for 15 min. Mix culture occasionally to ensure efficient cooling.
*Note: From this point forward, work quickly and keep cells ice-cold at all times. Never let cells warm and work in a cold room if necessary. Maintain sterility.*
    - Pour chilled culture into two sterilized 0.5 L centrifuge bottles and centrifuge cells at 4,000× *g* for 15 min at 4 °C.
    - Pour supernatant off without disturbing cell pellet. Wipe any residual media supernatant around the lip of the centrifuge bottle if necessary. Place centrifuge bottle with cell pellet immediately on ice.
    - Resuspend each cell pellet with 250 mL each of ice-cold 10% (v/v) glycerol solution (can be measured with the sterile graduated cylinder) by gentle swirling on ice. Be gentle with the cells, and DO NOT VORTEX. Keep cells cold at all times.
    - Once cells are evenly resuspended in 10% (v/v) glycerol, combine into one 0.5 L centrifuge bottle and centrifuge again at 4,000× *g* for 15 min at 4 °C.
    - After centrifuging, pour supernatant off without disturbing cell pellet. Place centrifuge bottle with cell pellet on ice.
    - Measure 250 mL of 10% (v/v) ice-cold glycerol in the same pre-chilled graduated cylinder. Resuspend cell pellet with this 250 mL of ice-cold glycerol solution by gentle swirling on ice. Again, DO NOT VORTEX and keep cells and centrifuge bottle cold at all times during the resuspension process.
    - Once cells are evenly resuspended in 10% (v/v) glycerol, centrifuge again at 4,000× *g* for 15 min at 4 °C.
    - Pour supernatant off without disturbing cell pellet. Place centrifuge bottle with cell pellet on ice.
    - Resuspend cell pellet with 30 mL of ice-cold glycerol solution by gentle swirling on ice. Again, DO NOT VORTEX and keep cells and centrifuge bottle cold at all times during the resuspension process. Transfer resuspended cells to a sterile 50 mL conical tube and top off conical tube to 50 mL total volume with ice cold 10% (v/v) glycerol.
    - Centrifuge again at 4,000× *g* for 15 min at 4 °C.
    - During this final centrifuge run, prepare a dry ice/ethanol bath as follows:
      In a small Styrofoam box, a pan, or dish-like container, add dry ice pellets and pour enough ethanol (or isopropanol) to cover the bed of dry ice. Place an empty 1 mL pipette tip rack on the bed of dry ice; the base of the pipette rack should be touching the ethanol but should not be submerged, such that when you place a 0.6 mL Eppendorf tube in the rack, the bottom half of the Eppendorf tube is suspended in the ethanol bath while the cap of the tube stays well above the ethanol.
    - When the final centrifuge run is complete, pour off or aspirate the supernatant. Resuspend the cell pellet gently with ~0.3 mL of 10% (v/v) glycerol into an even suspension and transfer to a sterile 15 mL conical tube. To resuspend the cells, you can use a 1,000 μL pipettor equipped with a pipette tip that has had ~3 mm cut off the end with a sterile razor blade. The reason for cutting off the end of the pipette tip is to widen the hole at the end to minimize shearing forces on your cells as you are pipetting them up and down to resuspend.
    - Once resuspended into an even cell suspension, measure OD_600_.
*Note: You will need make a ~1:500 dilution of the cells to get an accurate OD_600_ reading, e.g., 998 μL of water + 2 μL of cell suspension. Measure the OD_600_ of these diluted cells and then calculate OD_600_ of your actual cell suspension based on the dilution factor. For example, if your OD_600_ reading of your 1:500 dilution = 0.5, then the cell suspension is 0.5 × 500 = 250.*
    - The OD_600_ of your resuspended cells should be between 200 and 300 to achieve sufficient competency for triple plasmid transformations and expressions. If the OD_600 _is above 300, dilute the cells with cold 10% glycerol so that the final density lies between 200 and 300. If the OD_600_ is below 200, then centrifuge the cells again and resuspend in a smaller volume of 10% (v/v) glycerol, e.g., 0.1 mL instead of 0.3 mL, re-measure OD_600_, and adjust volumes as needed.
    - Pipette ~35 μL of cell suspension into a 0.6 mL Eppendorf tube and quickly place tube in the dry-ice/ethanol bath rack to freeze cells.
      The bottom of the Eppendorf tube should be submerged in the dry ice/ethanol bath, but do not let the cap of the tube come in contact with the ethanol; the alcohol will wick its way into the tube, ruining the cells.
    - Once cells are frozen (~2 min in the dry ice/ethanol bath), remove tube from bath, wipe the tube free of ethanol with a Kimwipe, and place the tube with frozen cells in a cryogenic freezer box containing dry ice.
    - Repeat until all cells have been aliquoted and frozen. Once all aliquots are frozen and in the box with dry ice, place box at -80 °C for long-term storage. You should obtain ~20–30 aliquots of competent cells from this procedure.
    - When done, place dry-ice/ethanol bath in a fume hood to allow dry ice to sublime. The ethanol/isopropanol can be poured into a bottle for re-use. **Caution:** the ethanol/isopropanol will off-gas CO_2 _for many hours, so if you pour it into a bottle be sure to leave cap lose/off to allow gas to escape, ensuring pressure does not build up inside the bottle.
- **Expression of protein with site-specifically encoded nhpSer**

- Fresh triple plasmid transformations must be performed for each expression. Never freeze expression cells containing plasmids; if expression cells are frozen with expression plasmids in them, the cells will grow in the presence of the correct antibiotics, but they will not express protein. Do not sequentially transform plasmids.
- To standardize this protocol in your lab, first run control expressions with sfGFP wt and sfGFP-150TAG to verify you can achieve the reference expression benchmarks (see Table 1 below) and ensure that nhpSer encoding at the TAG site is functioning as expected with a model protein. The protocol below outlines the protocol for a standard 50 mL test expression to encode nhpSer into sfGFP at position N150 (i.e., sfGFP-150TAG), as well as wild-type sfGFP. Pointers on how to scale up to a 1 L expression, as is often used for target proteins, are also included.
  1. Day 1: transformations
    - Prepare two LB/agar plates, one for sfGFP-wt and one for sfGFP-150TAG expressions, with antibiotics as follows:
      i. Sterilize LB/agar as described above. After autoclaving, allow to cool sufficiently to touch while still remaining liquid.
      ii. Pour 50 mL of LB/agar into a sterile 50 mL conical tube. Add 35 μL each of ampicillin, kanamycin, and spectinomycin stock solutions. Mix thoroughly and pour ~20–25 mL into each 100 mm plate. Final working concentrations of antibiotics should be 70 μg/mL ampicillin (for the pRBC plasmid), 35 μg/mL kanamycin (for the pERM2-nhpSer plasmid), and 70 μg/mL spectinomycin (for the pCDF-Frb-v1 plasmid).
      iii. Allow LB/agar to cool and solidify beside a flame with plate lid slightly ajar for 30 min.
      iv. Label the two plates accordingly, e.g., “sfGFP-WT” and “sfGFP-150TAG.”
    - Place two 1 mM electro-cuvettes on ice for 15 min to pre-chill.
    - Place two 0.6 mL Eppendorf tubes on ice and label, e.g., “sfGFP-WT” and “sfGFP-150TAG.”
      i. To the “sfGFP-WT” tube, add 2 μL each of pRBC-sfGFP WT, pERM2-nhpSer, and pCDF-Frb-v1 plasmids (~100–500 ng of each plasmid).
      ii. To the “sfGFP-150TAG” tube, add 2 μL each of pRBC-sfGFP-150TAG, pERM2-nhpSer, and pCDF-Frb-v1 plasmids.
      iii. Allow tubes to chill on ice for 5–10 min.
    - Thaw two aliquots of electrocompetent BL21(DE3) ∆*serC* cells. Cells can be thawed rapidly with the warmth of your fingers, but immediately place tube on ice once thawed.
    - Transfer 30 μL of electrocompetent BL21(DE3) ∆*serC* cells to each tube with plasmids and gently mix cells with plasmids by pipetting up and down 2–3 times. Cells should not appear stringy or behave like “snot,” which would indicate cells have lysed.
    - Transfer the “sfGFP-WT” electro-competent cell/plasmid mix into a pre-chilled electro-cuvette. Wipe metal electrode plates dry with a Kimwipe. Gently flick the cuvette to ensure cells are sitting in the bottom and stretch the full width of the cuvette.
    - Place electro-cuvette into the electroporator and electroporate according to instrument instructions for 1 mM cuvettes (e.g., 1,500 V for the Eppendorf Eporator).
*Note: The time constant should be 4–6 ms. If arc’ing occurs, discard cells and cuvette. Arc’ing occurs when DNA and/or cells have too much salt. Re-attempt electroporation by diluting a fresh cell/plasmid mix with equal volume of cold MQ water.*
    - Immediately add 1 mL of SOC media to electro-cuvette and resuspend cells. Transfer cells to a sterile 1.7 mL Eppendorf tube.
    - Repeat steps f–h for the “sfGFP-150TAG” electro-competent cell/plasmid mix.
    - Place both Eppendorf tubes in a shaker (250 rpm) and recover cells at 37 °C for 90 min.
*Note: Electro-cuvettes can be reused at least 10 times if washed shortly after use. Wash cuvettes as follows while cells are recovering:*
*i. MQ H_2_O.*
*ii. 0.1 M HCl (dilute acid helps to break down residual DNA in the cuvette).*
*iii. MQ H_2_O.*
*iv. 70% (v/v) ethanol.*
*v. Let dry upside down on a Kimwipe. Once dry, store with cap to maintain sterility.*
    - After 90 min of recovery, plate all the cells from the “sfGFP-WT” recovery culture onto the LB/agar/Amp/Kan/Spec plate labeled “sfGFP-WT.”
      To plate all the transformed cells, you cannot put the entire 1 mL of recovery culture on the agar plate as this is too much liquid and it will not dry properly. Instead, pellet the cells by centrifugation at 3,000× *g* for 3 min. Remove the top 900 μL of media and gently resuspend the pellet in the remaining ~100 μL. Spread this cell suspension evenly onto the agar plate.
    - Repeat plating process for the “sfGFP-150TAG” culture.
    - Let plates dry with lid partially open for ~20 min near a flame (maintaining sterility) and then incubate plates upside down overnight (~16 h) at 37 °C.
  2. Day 2: expressions
    - This protocol is intentionally written to avoid overnight liquid starter cultures that would reach stationary phase. Rather, in one day, you will use freshly grown colonies on an agar plate to grow a starter liquid culture in the morning, after which you will inoculate expression culture in the early afternoon. By the end of the day the cultures will be induced and allowed to express for the next 20–24 h. Having a large number (> 500) of colonies on each LB/agar plate is necessary to execute this one-day expression protocol.
    - All liquid cultures must contain 70 μg/mL ampicillin, 35 μg/mL kanamycin, and 70 μg/mL spectinomycin.
    - **Important:** Baffled culture flasks are critical to ensure adequate aeration of expression cultures.
    - Remove LB/agar plates from 37 °C incubator. There should be several hundred or thousands of colonies (examples of successful triple plasmid transformations are shown in Figure 3).
      **Figure 3.**
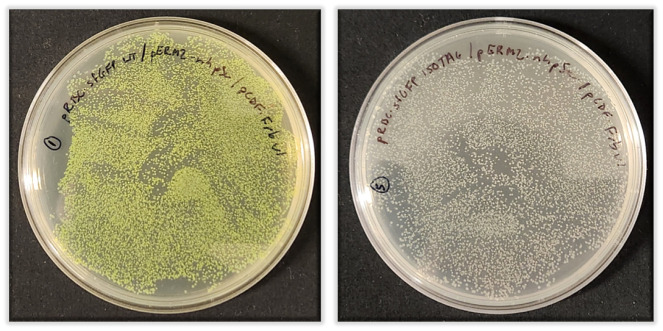
Representative LB/agar plates after triple plasmid co-transformation of pRBC-sfGFP WT (left) or pRBC-sfGFP-150TAG (right) with pERM2-nhpSer and pCDF-Frb-v1 plasmids and growth at 37 °C overnight (~18 h). The sfGFP-WT plates on the left are fluorescent due to leaky expression of the T7 expression system in BL21(DE3) cells. Note the size homogeneity of colonies and that all colonies on the pRBC-sfGFP WT plate are green and no green colonies appear on the pRBC-sfGFP 150TAG plate.
    - Prepare starter cultures
      i. Starter cultures are grown in *starter culture media* as defined above (2× YT + 0.5% (v/v) glycerol) with the same antibiotics and concentrations used in the LB/agar plates.
      ii. For small-scale (e.g., 50 mL) sfGFP control expressions, prepare two starter cultures by adding 5 mL of starter culture media (with ampicillin, kanamycin, and spectinomycin antibiotics) to 14 mL sterile culture tubes.
      iii. To inoculate these 5 mL cultures, scrape a “glob” of cells constituting several dozen to hundreds of colonies from overnight LB/agar plate with a sterile pipette tip, shake the glob off into the culture media, and break apart by gentle pipetting. Enough cells should be transferred to the 5 mL starter culture such that it is visibly turbid upon inoculation.
      iv. For larger scale expressions (e.g., > 1 L), prepare 50 mL of starter culture media (with antibiotics) in a baffled 250 mL culture flask. Transfer 5 mL of starter culture media onto the LB/agar plate containing transformed cells. Gently scrape all colonies with a sterile glass spreader to resuspend them into the liquid media. Transfer media with suspended cells into the 250 mL baffled culture flask containing ~45 mL of starter culture media. Add 1–2 drops of antifoam.
    - Grow starter cultures at 37 °C with shaking at 250 rpm for ~3–4 h until OD_600_ > 1. Do not grow overnight.
    - While starter cultures are growing, prepare expression media:
      i. For each 50 mL sfGFP test expression, add 5 mL of (room temperature) sterile 10× potassium phosphate buffer to 45 mL of 1.1× TB media in a 250 mL baffled culture flask. Add antibiotics and ~1–2 drops of antifoam.
      ii. For 1 L expressions, add 100 mL of sterile 10× potassium phosphate buffer to 900 mL of 1.1× TB media in a 2.8 L baffled Fernbach flask. Add antibiotics and ~5–6 drops of antifoam.
    - After ~3–4 h of starter culture growth, inoculate expression media as follows:
      i. For 50 mL of sfGFP test expressions, add 1 mL of the sfGFP-WT starter culture to 50 mL of TB expression media (in a baffled 250 mL culture flask). Repeat for the sfGFP-150TAG starter culture.
      ii. For a 1 L expression, add 10–20 mL of starter culture to each baffled Fernbach flask containing 1 L of TB expression media.
    - Grow expression cultures at 37 °C with shaking (200–250 rpm) until OD_600_ = 1.0. This should take approximately 3–5 h.
    - When cultures reach OD_600_ = 1, add IPTG to a final concentration of 0.5 mM.
      i. For 50 mL cultures, this corresponds to 50 μL of 0.5 M IPTG stock solution.
      ii. For 1 L cultures, this corresponds to 1 mL of 0.5 M IPTG stock solution.
    - Reduce temperature to 30 °C for sfGFP control expression cultures. For other target proteins, temperature can be reduced to as low as 20 °C. The Frb biosynthetic pathway functions optimally between 20 and 30 °C. Expression temperatures below 20 °C may be possible but expression times will need to be extended and overall yields will be diminished. Do not express target proteins above 30 °C since the Frb biosynthetic pathway does not function above this temperature.
    - For 50 mL expression cultures, add 1–2 more drops of antifoam. For 1 L expressions, add 5–6 more drops of antifoam.
    - Continue shaking cultures at 200–250 rpm for 20–24 h at the desired temperature (between 20 and 30 °C; optimal for sfGFP is 30 °C). During this expression period, the density of the culture (OD_600_) should increase significantly (up to 20).
  3. Day 3: Evaluating expression results and harvesting cells
**sfGFP control protein expression analysis**
    Approximately 20–24 h after induction, measure OD_600_ and fluorescence of both sfGFP and sfGFP-150TAG cultures using a fluorometer. Since sfGFP chromophore formation requires synthesis of full-length protein, fluorescence of whole cells provides a convenient strategy to evaluate the efficiency of sfGFP TAG codon suppression, and therefore nhpSer incorporation. Fluorescence can be measured with any fluorimeter capable of detecting sfGFP fluorescence (ex/em: 488/510 nm). Diluting the culture 1:10 to 1:100 in a buffer (e.g., 100 μL of culture + 1,900 μL of PBS for a 1:20 dilution) prior to fluorescence measurements may be necessary to obtain a signal within the linear range of the fluorometer.
**Test expression benchmarks:**
    In general, the sfGFP-150TAG culture fluorescence should be approximately 20%–30% that of the sfGFP wild-type culture, corresponding to 50–150 mg of sfGFP-150nhpSer per liter of culture (Table 1).
    The OD_600_ values will vary depending on target protein. Normal values will range from ~10 to 20 for sfGFP expressions. Final OD_600_ values below 5 are indicative of poor cell growth or toxicity due to target protein expression.
    **Table 1.** Benchmarks for sfGFP-WT and 150TAG protein expression yields, based on culture fluorescence

	Fluorescence of culture#	mg of sfGFP per liter culture*	OD_600_
wt sfGFP	8,030 (6,000–12,000)	458 (340–600)	10.0 (5–15)
sfGFP 150TAG (with Frb-v1 pathway)	2,550 (1,500–3,000)	120 (50–150)	17.0 (10–20)
    #The range indicated in parenthesis is considered normal depending on the day and reagent preparation. Fluorescence values reported here were obtained on a hand-held PicoFluor fluorometer (Turner Biosystems) by diluting cells directly from the culture into PBS (1:20). Fluorescence values are arbitrary and will depend on the fluorometer used. It is important that the relative ratio of sfGFP-150TAG and sfGFP-WT culture fluorescence is consistent with the above values. For reference, background cultures not expressing sfGFP have auto-fluorescence values of ~150–250.
    *Yield of sfGFP in milligrams per liter was determined using a fluorescence standard curve of purified sfGFP.
**Harvesting cells:**
    Harvest cells by centrifugation at 5,000× *g* for 15 min at 4 °C. Pour off culture supernatant and resuspend cells in an appropriate buffer.
    - The choice of buffer depends on the protein of interest, the downstream purification strategy, and the application, and should be determined by the user.
    - Adding a cryoprotectant [e.g., 10% (v/v) glycerol] to this buffer can help minimize adverse effects associated with freezing sensitive or unstable proteins. Cells can be flash frozen in liquid nitrogen and stored at -80 °C or you can proceed with purification.
    - For His_6_-tagged proteins to be purified via TALON resin, a recommended resuspension/lysis buffer would be 50 mM Tris pH 7.5, 500 mM NaCl, 10% (v/v) glycerol, 5 mM imidazole. Phosphatase inhibitors are not required since nhpSer is not hydrolyzed by phosphatases.

## Validation of protocol

For each expression, it is important to confirm faithful incorporation of nhpSer into the target protein. Several methods can be used to evaluate the phosphorylation status of the target protein.

1. Phos-tag electrophoresis: Phos-tag gel electrophoresis is a modified form of SDS-PAGE, in which phosphorylated proteins migrate with attenuated electrophoretic mobility compared to their non-phosphorylated counterparts. The degree to which protein migration is attenuated increases with sequential addition of phosphoryl groups, allowing one to distinguish between no, single-, and multi-pSer containing proteins (Kinoshita et al., 2006). Serendipitously, proteins with nhpSer migrate slower on Phos-tag electrophoresis than the same proteins with authentic pSer, allowing easy discrimination between the two [for examples, please see (Zhu et al., 2023) as well as Figure 4 below]. By measuring the density of the non-phosphorylated vs. pSer vs. nhpSer protein bands, one can estimate the percentage of expressed/purified protein that contains nhpSer. Important to note is that wild-type (non-phosphorylated) protein must be run side-by-side with the phosphorylated protein to observe relative shifts in electrophoretic mobility. Similarly, it is also helpful to express your target proteins with pSer as well so that you can confirm if the electrophoretic shifts of your nhpSer-containing protein are due to nhpSer encoding and not pSer [see our previous protocol for expressing proteins with pSer: (Zhu et al., 2022)]. Other advantages of Phos-tag electrophoresis include the ability to evaluate multiple samples at once, requiring only a standard SDS-PAGE electrophoresis setup, and being economical. Do not run molecular weight markers on Phos-tag gels, as they often contain EDTA, which is not compatible with Phos-tag. See Figure 4 below for an example of SDS-PAGE and Phos-tag gels of nhpSer and pSer containing sfGFP.
*Note: The attenuated electrophoretic mobility of sfGFP-150pser/nhpSer proteins in Phos-tag gels are particularly pronounced and easily discernable; however, the degree to which other phosphorylated proteins migrate slower than their non-phosphorylated counterparts depends on the protein and the site of encoding. If you do not see a convincing electrophoretic shift between your phosphorylated and non-phosphorylated proteins, consider optimizing the Phos-tag electrophoresis procedure to improve resolving power by (i) increasing concentration of the Phos-tag acrylamide reagent from 25 or 50 μM to 100 μM or higher, (ii) decreasing the amount of protein loaded for narrower bands and enhanced resolution between similarly migrating protein bands, (iii) decrease the total acrylamide concentration [e.g., from 15% to 10% (w/v) acrylamide] so that target proteins bands travel at least half-way through the gel thereby increasing separation, and (iv) run sfGFP WT/150nhpSer control proteins alongside the proteins of interest to ensure the Phos-tag gel is run correctly. If still no shift is observed on Phos-tag electrophoresis, you will have to confirm incorporation via mass spectrometry (see below).*
  **Figure 4.**
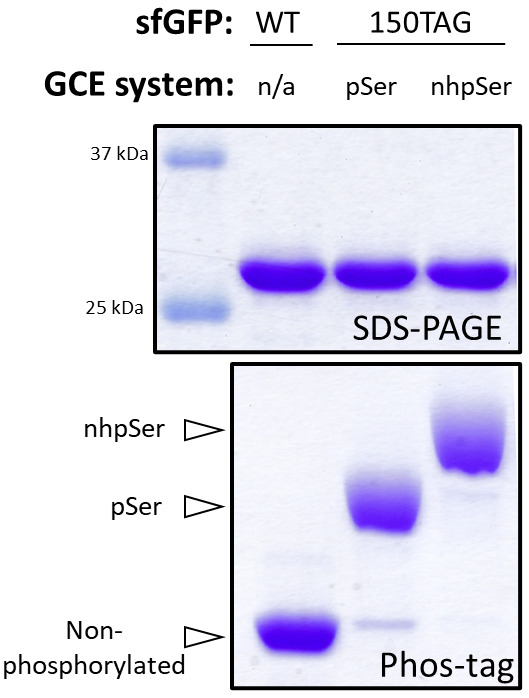
Assessing nhpSer encoding into sfGFP by Phos-tag gel electrophoresis. SDS-PAGE (top) and Phos-tag (bottom) gels of purified WT sfGFP, sfGFP-150pSer, and sfGFP-150nhpSer proteins. The sfGFP-150pSer protein was produced following our prior protocol (Zhu et al., 2022) and the sfGFP-150nhpSer protein was produced using the protocol described here. Because they migrate differently on Phos-tag gels, non-phosphorylated, pSer-containing, and nhpSer-containing proteins can be resolved from one another, and the fraction of each form can be easily evaluated in each sample. In this example, the sfGFP-150pSer protein sample is > 90% phosphorylated with pSer, with only trace amounts of non-phosphorylated protein that migrates identically as the wild-type sfGFP protein, while the sfGFP-150nhpSer protein is > 95% phosphorylated with nhpSer, with only trace amounts of pSer-containing contaminant protein and no detectable non-phosphorylated protein. Note that all proteins migrate identically on SDS-PAGE, indicating the electrophoretic shifts observed in Phos-tag are due to phosphorylation status and not differences in protein size. These samples were incubated at 95 °C for 5 min in standard Laemmli buffer prior to their loading on gel. The Phos-tag gel contained 50 μM Phos-tag acrylamide, 100 μM MnCl_2_, and a 29:1 acrylamide:bis-acrylamide ratio and was run at 120 V for 1.5 h at room temperature.
2. Mass spectrometry: whole-protein mass spectrometry (MS) is the most convincing methodology for confirming nhpSer encoding, if facilities are available and the target protein is amenable to whole-protein MS analysis. Note that some mass spectrometers may not have sufficient resolution to distinguish masses of whole proteins with pSer and nhpSer (∆ = 1.98 Da), in which case Phos-tag electrophoresis or tryptic-digests followed by MS/MS fragmentation (i.e., bottom-up analysis) can be used. For an example of high-resolution whole-protein MS data distinguishing between sfGFP with pSer and nhpSer, please see Figure 3 in Zhu et al. (2023). When analyzing whole-protein MS data of sfGFP proteins, be sure to account for chromophore maturation, which results in the loss of a water molecule and two protons; therefore, the mass will be exactly 20 Da less than that predicted by amino acid sequence.
*Important note: Tryptic-digest followed by MS fragmentation analysis (i.e., “bottom-up” MS/MS analysis) of nhpSer-proteins is useful for confirming nhpSer incorporation and the site of incorporation, but it should not be used to make conclusions regarding the degree of nhpSer encoding.*
3. Western blotting: a variety of antibodies are available to detect specific pSer-containing proteins via western blotting. We have not yet tested if such pSer-specific antibodies also cross-react with the same proteins containing nhpSer but expect this to be dependent on the protein, site of phosphorylation, and the antibody. Note that while western blotting with a phosphorylation-sensitive antibody may be useful to confirm target protein phosphorylation, it cannot be used to evaluate the extent of phosphorylation without running alongside a standard curve of homogenously phosphorylated protein standard. Alternatively, it is possible to image Phos-tag gels via western blotting to evaluate the degree of wild-type, pSer, and nhpSer-containing target protein in a mixture of other proteins [e.g., see Supporting Information Figure S26 in Zhu et al. (2023)]. In these cases, using an antibody that binds to its epitope irrespective of protein phosphorylation status is important (e.g., FLAG or MYC tags).

## General notes and troubleshooting

**General notes**

1. Truncation. Like most standard *E. coli* expression hosts, the BL21(DE3) ∆*serC* strains contains Release Factor-1 (RF1), the protein responsible for terminating translation at TAG codons. Consequently, nhpSer encoding at TAG codons is in constant competition with RF1 and so a mixture of full-length protein containing nhpSer and prematurely truncated protein will be produced. Keeping this in mind when designing the expression construct of the target protein is important. Appending a C-terminal purification tag ensures one purifies only full-length protein, though not all proteins express well with C-terminal tags. If an N-terminal purification tag is used, the truncated protein may likely co-purify with full-length protein, and could be the dominant purified protein species. In these cases, you will need to develop additional strategies to separate the truncated protein. Note that if the target protein is a homo-oligomer, truncated protein may be difficult or impossible to purify away when the site of encoding is C-terminal to the oligomerization domain, as both truncated and full-length proteins will oligomerize and co-purify regardless of where the purification tag is placed.
2. Compatible plasmids for expressing target protein. In this protocol, three plasmids must be present in the cell in order to encode nhpSer into a target protein. For multiple plasmids to be faithfully maintained in *E. coli*, each plasmid must have an origin-of-replication from a different *compatibility group*, as well as confer different antibiotic resistances. We engineered the pRBC vector (ampicillin resistance, p15a origin-of-replication) to meet these requirements when being propagated with the nhpSer machinery vector (pERM2-nhpSer, kanamycin resistance with pUC origin-of-replication) and the nhpSer biosynthetic pathway vector (pCDF-Frb-v1, spectinomycin resistance with CloDF origin-of-replication). Critical to note is that because the pERM2-nhpSer vector harbors a pUC origin of replication, you *cannot* use traditional pET, pGEX, or pBAD-like vectors to express target proteins because these vectors harbor an origin-of-replication from the same compatibility group as the pERM2-nhpSer vector (the pUC origin of replication is a derivative of pBR322/pMB1 origins and is therefore in the same compatibility group). The pRBC vector was also engineered to be compatible with established pSer GCE encoding systems, which use the pKW2-EFSep machinery plasmid (chloramphenicol resistance and pBR322 origin-of-replication) enabling the flexibility to encode either pSer or nhpSer with the same pRBC vector depending on downstream applications. If you wish to use a vector other than pRBC to express target proteins with pSer or nhpSer, ensure that it has a p15a or RSF origin-of-replication and that it confers resistance to an antibiotic other than kanamycin, spectinomycin, and chloramphenicol (e.g., ampicillin, tetracycline).

**Troubleshooting**

1. Construct design. There are a variety of reasons why a target protein may not express when encoding nhpSer (or pSer), even though your sfGFP-150TAG controls are working well and your equivalent wild-type target protein construct expresses at high yields. In these situations, it is important to consider that phosphorylation is a *post*-translational modification, whereas with GCE systems the phospho-amino acids are installed *during* translation, and we therefore require the target protein to fold properly with the phospho-amino acid present and while isolated from any potential interacting/stabilizing partner proteins. So, even when using highly efficient pSer/nhpSer GCE systems to encode phospho-amino acids into otherwise well-behaved, highly expressing and soluble native proteins, there may be difficult-to-anticipate challenges that will require clever strategies to coax target phospho-proteins to express stably and efficiently. Strategies to improve phospho-protein expression, folding and stability include (i) fusing solubility tags to the N- and/or C-terminus of the target protein (e.g., SUMO, GST, MBP), (ii) co-expressing folding chaperone proteins (e.g., GroEL/ES, Trigger Factor), and (iii) co-expressing binding partner proteins, if they are known. Similarly, fusing a cleavable fluorescent reporter protein (e.g., super-folder GFP) to the C-terminus of your target protein provides a convenient strategy to screen for optimal expression conditions by measuring cell fluorescence. In this case, only when nhpSer is encoded and full-length target protein is produced will translation continue into the sfGFP causing the cells to fluoresce. Including a cleavage site between target protein and sfGFP (e.g., TEV, 3C) allows the sfGFP to be removed should it interfere with function or downstream applications.
2. Transformations. This protocol requires three plasmids to be transformed into *E. coli* cells simultaneously for every expression, and with an efficiency that allows you to obtain enough cells to inoculate a starter culture that only takes ~1/2 a day to grow to a density sufficient to inoculate expression cultures. If only a few colonies are obtained after the transformation, you will not have enough cells to inoculate your starter culture, and then this starter culture will take too long to grow. Overnight growth of the starter culture would seem an obvious strategy to overcome this issue; however, we have observed poor expression of nhpSer proteins when starter cultures are allowed to reach stationary phase. Protein also will not express if expression cells containing plasmids were grown from a frozen glycerol stock as opposed to from freshly transformed cells. If you are experiencing low transformation efficiencies, this may be caused by (i) poorly prepared electrocompetent cells, (ii) too low plasmid concentrations, (iii) degraded, compromised, or salty plasmids (see note below) and/or (iv) improper electroporator parameters. To troubleshoot the source of the inefficiency, transform each plasmid individually and plate on LB/agar plates with a single antibiotic (ampicillin, kanamycin, and spectinomycin). You should obtain a “lawn” of cells for each individual plasmid transformation. If one of the plasmids transforms notably worse than another, there may be integrity issues associated with that particular plasmid. If you get few colonies on all three single plasmid transformations, either the electrocompetent cells were prepared incorrectly or the electroporator is not functioning properly.
3. Plasmid integrity. We have observed in rare cases the pERM2-nhpSer plasmid recombining when propagated in *E. coli* cells to render it non-functional. We recommend sequencing the plasmid when you receive it from Addgene and after propagation to ensure integrity. Economical, whole-plasmid sequencing is available from a variety of companies, such as Plasmidsaurus. Sequencing the pCDF-Frb-v1 and pRBC-sfGFP-150TAG plasmids are also recommended, though we have not observed any stability concerns with these plasmids when propagated in DH10b *E. coli* cells.
